# Dopamine and the dynamics of subthalamic and leg muscle activities in parkinsonian stepping

**DOI:** 10.1093/brain/awaf464

**Published:** 2025-12-13

**Authors:** Thomas G Simpson, Shenghong He, Laura Wehmeyer, Alek Pogosyan, Fernando Rodriguez Plazas, Ashwini Oswal, Michael G Hart, Rahul S Shah, Harutomo Hasegawa, Christoph Wiest, Sahar Yassine, Xuanjun Guo, Philipp A Loehrer, Anca Merla, Pablo Andrade, Veerle Visser-Vandewalle, Andrea Perera, Kenneth Adindu, Ahmed Raslan, Andrew O’Keeffe, Marie-Laure Welter, Francesca Morgante, Keyoumars Ashkan, Erlick A Pereira, Huiling Tan

**Affiliations:** Medical Research Council Brain Network Dynamics Unit, Nuffield Department of Clinical Neurosciences, University of Oxford, Oxford OX1 3TH, UK; Medical Research Council Brain Network Dynamics Unit, Nuffield Department of Clinical Neurosciences, University of Oxford, Oxford OX1 3TH, UK; Medical Research Council Brain Network Dynamics Unit, Nuffield Department of Clinical Neurosciences, University of Oxford, Oxford OX1 3TH, UK; Medical Research Council Brain Network Dynamics Unit, Nuffield Department of Clinical Neurosciences, University of Oxford, Oxford OX1 3TH, UK; Medical Research Council Brain Network Dynamics Unit, Nuffield Department of Clinical Neurosciences, University of Oxford, Oxford OX1 3TH, UK; Medical Research Council Brain Network Dynamics Unit, Nuffield Department of Clinical Neurosciences, University of Oxford, Oxford OX1 3TH, UK; City St George’s, University of London & St George’s University Hospitals NHS Foundation Trust, Neuroscience & Cell Biology Research Institute, London EC1V 0HB, UK; City St George’s, University of London & St George’s University Hospitals NHS Foundation Trust, Neuroscience & Cell Biology Research Institute, London EC1V 0HB, UK; Department of Neurosurgery, King’s College Hospital, London SE5 9RS, UK; Medical Research Council Brain Network Dynamics Unit, Nuffield Department of Clinical Neurosciences, University of Oxford, Oxford OX1 3TH, UK; Medical Research Council Brain Network Dynamics Unit, Nuffield Department of Clinical Neurosciences, University of Oxford, Oxford OX1 3TH, UK; Medical Research Council Brain Network Dynamics Unit, Nuffield Department of Clinical Neurosciences, University of Oxford, Oxford OX1 3TH, UK; Medical Research Council Brain Network Dynamics Unit, Nuffield Department of Clinical Neurosciences, University of Oxford, Oxford OX1 3TH, UK; Department of Neurology, Philipps-University Marburg, Marburg 35043, Germany; Department of Neurosurgery, King’s College Hospital, London SE5 9RS, UK; Department of Stereotactic and Functional Neurosurgery, University of Cologne, Cologne 50937, Germany; Department of Stereotactic and Functional Neurosurgery, University of Cologne, Cologne 50937, Germany; Department of Neurosurgery, King’s College Hospital, London SE5 9RS, UK; Department of Neurosurgery, King’s College Hospital, London SE5 9RS, UK; Department of Neurosurgery, King’s College Hospital, London SE5 9RS, UK; Department of Neurosurgery, King’s College Hospital, London SE5 9RS, UK; Department of Neurophysiology, CHU Rouen, Rouen University, Rouen 76000, France; City St George’s, University of London & St George’s University Hospitals NHS Foundation Trust, Neuroscience & Cell Biology Research Institute, London EC1V 0HB, UK; Department of Neurosurgery, King’s College Hospital, London SE5 9RS, UK; City St George’s, University of London & St George’s University Hospitals NHS Foundation Trust, Neuroscience & Cell Biology Research Institute, London EC1V 0HB, UK; Medical Research Council Brain Network Dynamics Unit, Nuffield Department of Clinical Neurosciences, University of Oxford, Oxford OX1 3TH, UK

**Keywords:** Parkinson’s disease, stepping, subthalamic nucleus (STN), dopamine, deep brain stimulation (DBS), local field potentials (LFPs)

## Abstract

Freezing of gait (FOG) is a devastating symptom of Parkinson’s disease (PD) often resulting in disabling falls and loss of independence. It affects half of patients, yet current therapeutic strategies are insufficient, and the underlying neural mechanisms remain poorly understood.

This study investigated beta oscillation dynamics in the subthalamic nucleus (STN) during different movement states (sitting, standing and stepping), while examining the effects of levodopa. Specifically, it aimed to identify pathological activity during stepping by analysing the relationship between the STN and leg muscles and how this is modulated by levodopa. Local field potentials (LFPs) in the STN and leg muscle activity measured as EMG of the gastrocnemius and peroneus longus were recorded in 14 PD patients during sitting, standing and stepping, ON and OFF levodopa.

Levodopa reduced stepping frequency variability, implying improved stepping rhythmicity. Low-beta (12–20 Hz) and high-beta (21–35 Hz) were differentially modulated by stepping movements and levodopa, with reduced high-beta and increased low-beta during stepping compared with standing and sitting. In contrast, levodopa reduced low-beta but increased high-beta activity, highlighting a potential physiological function of high-beta in the STN. Additionally, step-phase-specific effects of levodopa were observed including reduced broad-beta band activity in the STN and leg muscles during the late stance and lift-off phase of the contralateral leg when ON medication. Furthermore, STN beta bursts were associated with increased muscle activation at movement initiation, potentially reducing the ability to move freely. This study observed different effects of movement status (sitting versus stepping versus standing) on the average amplitude of low- versus high-beta frequency bands, suggesting they may serve distinct functional roles. Furthermore, there is a step-phase-specific effect of levodopa on STN LFPs, EMGs and intermuscular coherence during stepping. These findings offer insight for developing phase-specific stimulation strategies targeting STN beta oscillations during gait.

## Teaser

Simpson *et al.* reveal step phase-specific effects of levodopa on STN beta and leg muscle activities, as well as distinct roles for low- and high-beta activity in the STN. These findings advance our understanding of gait pathophysiology in Parkinson’s disease and support development of phase-specific DBS strategies.

## Introduction

Freezing of gait (FOG) is a severely debilitating symptom of Parkinson’s disease (PD) affecting around 70% of patients when disease duration exceeds 10 years.^1^ This symptom often leads to a significant loss of independence, a reduction in quality of life and an increased risk of disabling falls. Current interventions for Parkinsonian gait generally offer limited efficacy,^2,3^ highlighting the demand for optimized therapeutic approaches. Better understanding of the neural basis of gait control in PD may offer new insight on improving the treatment of gait difficulties, including FOG.

The subthalamic nucleus (STN) has a key role in gait, as it receives ‘hyperdirect’ inputs from motor areas and serves as an important moderator of basal ganglia output.^4^ It is thought to regulate the integration of cortical and cerebellar information by activating or inhibiting the mesencephalic locomotor region (MLR),^5,6^ which has an active role in initiating and modulating spinal neural circuitry for motor control.^7^ As the primary surgical target for deep brain stimulation (DBS) in PD, the STN has been extensively studied via electrophysiological recordings and consistently shown to exhibit increased oscillatory activity in the beta (13–30 Hz) frequency band, which has been linked to symptoms of rigidity and bradykinesia.^8,9^ This overactivity can lead to excessive inhibition of the MLR, disrupting the normal initiation and regulation of gait, and potentially contributing to symptoms such as FOG episodes.^10^ In addition, prolonged STN beta burst durations during gait have been shown to differentiate freezers from non-freezers, with shorter bursts observed in non-freezers.^11^ Further examination of STN beta modulation within the gait cycle has revealed step-phase-specific fluctuations, possibly reflecting variations in motor output.^12^

Dopaminergic treatment with levodopa has been consistently shown to decrease beta activity in subcortical structures, including the STN,^13,14^ while also improving objective measurements of gait such as velocity, stride length, rigidity and movement initiation.^15-17^ However, the effect of levodopa on gait-phase-related beta-band modulation in the STN remains unclear. Similarly, DBS of the STN, which is known to attenuate beta activity, has demonstrated efficacy in improving PD motor symptoms, including gait and FOG.^18^ Recent advancements in adaptive DBS based on beta activity have further shown promise in ameliorating gait symptoms.^19,20^ Nevertheless, the impact of DBS on gait in PD is complex, with most studies reporting improvements but occasional cases observing worsened or newly induced FOG.^21^

Building on these insights, other studies have focused on the distinct functional roles of STN activity in the low- versus high-beta frequency range,^22^ providing further understanding of gait regulation and the pathophysiology of FOG. Low-beta activity (13–23 Hz) has been reported to be modulated by levodopa and to strongly correlate with bradykinesia and rigidity.^22,23^ In contrast, patients with FOG exhibited higher power in the high-beta band, which was significantly reduced by levodopa and associated with suppression of FOG.^24^ Recent research also suggests that low-beta and high-beta cortico-STN coherence arises through distinct networks, possibly reflecting indirect and hyperdirect pathways, respectively.^25,26^ Meanwhile, the beta desynchronization during lower limb movements is characterized by a greater involvement of higher-beta frequencies (24–31 Hz) compared with upper limb movements.^27^ In addition, STN activity that is rhythmically modulated by the stepping phase is also focused on the high-beta frequency range.^12^ These prior findings underscore the critical role of STN beta oscillations, in particular the high-beta activities in gait regulation and FOG control, emphasizing the need for a deeper understanding of these mechanisms to optimize therapeutic strategies.

Apart from abnormal neural activities, pathological muscle activation patterns in the lower limbs have also been linked to FOG.^28-30^ For example, FOG is associated with a reduction in total EMG activity in lower limb muscles, including the tibialis anterior (TA) and gastrocnemius (GA), as well as shorter durations of muscle activation.^28-30^ This reduction was accompanied by increased amplitudes of EMG bursts in the TA, suggesting a compensation strategy of pulling the leg into swing. More recent research using frequency domain analysis of EMG indicates that PD freezers exhibit increased muscle activity within the alpha and low-beta bands in both the TA and GA muscles compared with healthy controls during walking.^31^ In addition, beta bursts in the cortical motor network have been found to be associated with increased beta band activity in upper limb muscles and cross-muscle phase synchrony in healthy motor control.^32,33^ This suggests that motor control relies on coordinated brain–muscle interactions, where disruptions in such synchronization may contribute to pathological states in PD. In fact, increases in pathological beta and theta rhythms in the STN have been observed to precede a temporal chain of abnormal lower limb muscle firing detected by EMG.^34^ Nevertheless, the precise mechanisms by which abnormal brain activity translates into pathological muscle activation, and how this contributes to gait impairments in PD, is poorly understood.

This study examines the dynamics of STN and EMG activities during stepping in PD, and how they are modulated by dopaminergic medication. Stepping-in-place is used in this study as a simplified yet functionally relevant motor behaviour that captures core elements of locomotion. Stepping-in-place minimizes movement-related artefacts that often confound electrophysiological recordings during free walking, allowing for more precise characterization of neural activity associated with gait. Additionally, stepping-in-place can be performed in constrained environments, increasing experimental control and reproducibility across sessions. STN local field potential (LFP) recordings were simultaneously monitored with the activity of the gastrocnemius (GA) and peroneus longus (PL) muscles to investigate how abnormal neural activity translates into pathological muscle activation. GA is a key muscle in stance limb stability and has been implicated in altered activity and coherence patterns during FOG episodes.^30,35,36^ PL, as a major mover and stabilizer of the foot and ankle, acts complementary to GA during stepping, especially in the stance phase.^37^ This muscle selection enables investigation of muscle coupling dynamics during stepping and their potential modulation by pathological brain activity. It was previously shown that sensorimotor cortical beta bursts are associated with increased muscle activation and intermuscular coherence in the beta frequency band.^33^ Based on this finding, it was hypothesized that a similar relationship might exist between STN beta bursts and coupling between lower limb muscles. It is further speculated that elevated muscle co-activation, especially during movement phases when the muscles are normally expected to act in opposition, may reflect altered or maladaptive motor control strategies.

This study shows that levodopa differentially influenced low- and high-beta activities in the STN during standing and stepping, providing insights into the distinct roles of beta oscillations in these movement states. Furthermore, this work aims to better describe how STN beta activity and lower extremity muscle activity dynamically change within the step cycle and how these changes are modulated by levodopa at different stepping phases. In addition, the relationship between pathological STN beta bursts and aberrant muscle activation patterns during stepping is explored. The ultimate objective is to propose a step-phase-specific stimulation strategy to directly target stepping and gait deficits in PD.

## Materials and methods

### Ethical approval

This experiment was approved by the South Central–Oxford C Research Ethics Committee.

### Patients

A total of 14 patients were recruited for the study {age = 64.5 ± 5.1 [mean ± standard deviation (SD)] years, disease duration = 11 ± 6.5 (mean ± SD) years}. Written informed consent was obtained in line with the Declaration of the Principles of Helsinki. Patients were able to understand and complete the task, although in some cases symptoms were so debilitating, especially when OFF medication, that the paradigm could not be executed in full. In these instances, patients performed for the maximum duration possible. Recordings were conducted 4 to 7 days after the first surgery, which involved bilateral implantation of DBS electrodes in the STN (see the ‘Recordings’ section). Patient data, including the surgical target, age, sex assigned at birth, disease duration and predominant symptoms, are reported in Table 1. Patient levodopa equivalent daily dose and Unified Parkinson's Disease Rating Scale part III (UPDRS-III) score (ON and OFF medication) are reported in Table 2.

**Table 1:** Patient data

Patient ID	DBS leads	Target	DH	M/F	Age (years)	Dis. dur (years)	Predominant symptoms before surgery	Excluded data: ON (%)	Excluded data: OFF (%)
P001	Medtronic SenSight (directional)	STN	R	F	63	9	Akinetic-rigid	0.98	0.48
P002	Medtronic SenSight (directional, Percept)	STN	L	M	75	6	Tremor both hands, light rigidity	6.43	4.31
P003	Medtronic SenSight (directional)	STN	R	M	61	5	Tremor, worst on right side	12.23	7.22
P004	Medtronic SenSight (directional)	STN	–	M	58	12	Rigidity and bradykinesia	4.21	1.80
P005	Boston Scientific (directional)	STN	R	M	66	10	Akinetic-rigid	5.68	2.64
P006	Medtronic SenSight (directional)	STN	R	M	57	5	Tremor dominant worst on right, bradykinesia and FOG	3.59	4.51
P007	Boston Scientific (directional)	STN	R	M	63	13	Akinetic-rigid	85.92	84.75
P008	Boston Scientific Octrode (non-directional, 2201 leads)	Vim/Vop + STN	R	M	70	9	Tremor dominant	23.51	30.07
P009	Medtronic SenSight (directional, 33005 leads)	STN	R	F	62	12	Akinetic-rigid	10.88	11.44
P010	Boston Scientific (directional)	STN	R	M	69	30	Tremor dominant	6.82	18.49
P011	Medtronic SenSight (directional)	STN	R	F	67	12	Severe OFF symptoms with dyskinesia and rigidity, some right-side tremor	0.21	3.37
P012	Medtronic SenSight (directional)	STN	R	M	67	6	Tremor and rigidity in left side	13.58	7.21
P013	Boston Scientific (directional)	STN	R	M	61	14	Tremor dominant	23.92	42.06
P014	Medtronic SenSight (directional)	STN	–	F	68	7	Rigidity in left side	1.14	0.90

The patient ID is given, along with the type of deep brain stimulation (DBS) leads, and the surgical target. The patients’ dominant hand (DH) is also shown (R and L for right and left, respectively), as is their sex assigned at birth (M/F), age, disease duration (Dis. dur) and predominant symptoms before surgery. FOG = freezing of gait; STN = subthalamic nucleus; Vim = ventralis intermedius; Vop = ventralis oralis posterior.

**Table 2:** Patient data continued

Patient ID	Medication levodopa equivalent daily dose		UPDRS-III		Bipolar recording configuration		Chronic stimulation configuration		Freezer
	Before ext.	During ext.	ON med.	OFF med.	Left	Right	Left	Right	
P001	1260 mg	Same	12	52	3–6	11–14	1.9 mA, case +, 4 −, 5 −, 6 −	1.6 mA, case +, 9 −, 10 −, 11 −	No
P002	800 mg	Same	49	51	1–2	9–10	2.5 mA, case +, 0 −	3.0 mA, case +, 8 −	No
P003	1150 mg	Same	11	22	4–7	14–15	3.8 V, case +, 1 −	2.1 V, case +, 9 −	No
P004	1555 mg	Same	10	47	2–5	13–15	3.8 V, case +, 1 −	3.8 V, case +, 10 −	No
P005	900 mg	Same	47	67	3–6	8–11	3.6 mA, (case +, 3 −) or 3.1 mA (case +, 0 −)	1.9 mA, case +, 9 −	Yes
P006	1230 mg	Same	12	67	1–4	13–15	3.0 V, case +, 0 −	2.8 V, case +, 11 −	No
P007	1100 mg	900 mg	29	43	6–7	9–12	2.0 mA, case +, 4 – (10%), 1 – (90%)	2.8 mA, case +, 12 – (20%), 10 – (80%)	Yes
P008	400 mg	Same	15	21	4–5	9–10	4.1 mA, case + 16%, 0 − 40%, 1 + 2%, 2 + 13%, 3 − 45%, 4 − 14%, 5 + 8%, 6 − 1%, 7 + 1%	4.6 mA, case + 89%, 8 − 57%, 9 + 6%, 10 + 2%, 11 − 5%, 12 − 38%, 13 + 3%	No
P009	740 mg	Same	6	31	0–1	8–10	2.9 mA, 1 − (1.0), 0 − (1.0), case +	2.0 mA, 11 +, 12 −, 13 −, 14 −	No
P010	700 mg	Same	31	37	0–1	8–9	2.3 mA, case +, 0 −	0.6 mA, case +, 10 − (25%), 9 − (25%), 8 − (50%)	No
P011	1103 mg	Same	9	57	1–4	12–15	0.6 mA, case +, 2 −	0.6 mA, case +, 10 −	No
P012	475 mg	Same	30	24	1–4	11–14	1.6 mA, case +, 4 − (0.6 mA), 5 − (0.6 mA), 3 − (0.4 mA)	2.7 mA, case +, 13 − (0.6 mA), 14 − (1.5 mA), 15 − (0.6 mA)	No
P013	980 mg	Same	17	45	2–5	9–12	2.2 mA, case +, 4 − (23%), 5 − (54%), 6 − (23%)	1.5 mA, case +, 12A − (31%), 13 − (46%), 14 − (23%)	Yes
P014	745 mg	Same	17	24	2–5	12–15	0.5 mA, case +, 2 −	0.5 mA, case +, 10 −	Yes

Medication before and after externalization is reported, along with the Unified Parkinson’s Disease Rating Scale part III (UPDRS-III) both ON and OFF medication. The bipolar configuration used for the analysis, as well as the configuration for chronic stimulation, are reported. Contacts are numbered from 0 on the left hemisphere and from 8 on the right. For directional leads, contacts 1–3 (left) and 9–11 (right) correspond to the directional contacts on the second-lowest level. Whether or not the patient is a freezer is also included. Pat. = patient; ext.= externalization; med.= medication.

### Experimental set-up

The paradigm consisted of three separate segments: resting (sitting), standing and stepping. In the rest condition, patients adopted a seated posture with their eyes open for 2 min. In the standing condition, patients alternated between 1 min of upright posture on the force plates and 1 min of seated posture, repeated five times for a total of 5 min standing. Similarly, in the stepping condition, patients alternated between stepping in place (while on the force plates) for 1 min and seated posture for 1 min, also repeated five times for a total of 5 min stepping. During the stepping-in-place condition, patients were instructed to step at a comfortable and consistent frequency, and to imagine themselves walking normally. As the focus of the study was on stepping performance rather than sit-to-stand transitions, hand support was offered to most patients during the brief transitions between seated and upright postures to ensure safety. A minute typically refers to an artefact-minimal (see the ‘Stepping phase-related modulations’ section) period of 1 min, confirmed visually to mitigate the effect of movement artefacts. All 1-min segments of standing and stepping included in the analysis were performed without hand support, except for one particularly frail patient who required support during stepping and standing. To synchronize behavioural conditions with the electrophysiological data, the conditions were recorded in separate files (resting, standing and stepping). Furthermore, the movement status of the patient was interpreted from the force plates.

The paradigm was performed twice: once in the OFF medication state and once in the ON medication state. The OFF condition was completed in the morning, with patients’ normal doses of levodopa withheld overnight. The ON condition was completed in the afternoon, with patients’ normal doses of levodopa administered at midday. This enabled a direct comparison between the two states.

### Recordings

The surgical target was the STN. DBS systems from two companies were implanted: Medtronic Inc. Neurological Division (octopolar directional leads, SenSight^TM^ model 33005) or Boston Scientific (octopolar directional leads, Vercise^TM^ model DB-2202). Electrodes were implanted as previously described,^38^ connected to temporary lead extensions and externalized through the temporal or frontal scalp. LFPs from the STN were recorded throughout the paradigm using the TMSi-SAGA amplifier (TMSi), at a sampling frequency of 4096 Hz. EMGs were simultaneously recorded using the same amplifier with the electrodes placed on four separate locations in bipolar configuration: left gastrocnemius (GA_L_), left peroneus longus (PL_L_), right gastrocnemius (GA_R_), right peroneus longus (PL_R_). The gastrocnemius was selected due to its significant role in gait, such as influence on speed and power, propulsion and control of important joints, with a primary role in stance limb stability.^35^ Altered timing and activation patterns of the gastrocnemius have been previously implicated in FOG.^36^ The peroneus longus was chosen for its key role in foot and ankle stability, which is critical for propulsion, balance and postural control.^37,39^ Additionally, two force plates were recorded using the same system (recorded at the same sampling frequency) in order to capture the phase of the steps. The ground electrode was placed on the patients’ wrist.

### Contact selection

Bipolar configuration was applied to LFP recordings in post-analysis to reduce activities from volume conduction and to focus on locally generated activities. Several different bipolar configurations were created *post hoc* based on unipolar LFP recordings from neighbouring contacts. The combinations tested for directional leads were 1–2, 1–3, 1–4, 2–5, 3–6, 4–7, 5–8, 6–8, 7–8, while for non-directional leads 1–2, 2–3, 3–4, 4–5, 5–6, 6–7, 7–8 were tested. Please note, a single contact was not created by combining numerous directional contacts. Instead, directional and ring contacts were used individually to form bipolar configurations. From these, continuous wavelet transform (CWT) was utilized to determine the amplitude spectral density (ASD) of all bipolar signals from the OFF medication rest condition. The bipolar configuration with the largest beta activity was selected as the configuration for use in the analysis. In 50% of the tested hemispheres (14 out of 28), one of the contacts (in the bipolar configuration with the largest beta amplitude) was used in the chronic stimulation configuration. The selected bipolar configuration is reported in Table 2, along with the final chronic stimulation configuration.

### Data processing

The following analysis pipeline was implemented primarily in MATLAB (version 2019b). R version 4.4.2 was used for ANOVA, and Spike2 for visualization.

#### Stepping analysis

Stepping frequency was defined as the number of steps completed per second. This was calculated by counting the number of steps (by observing the number of times the amplitude of the force plate exceeded a threshold based on the individual patient’s stepping force) during the artefact-free part of the trial (see the ‘Trial rejection’ section) and dividing this value by the number of seconds. This was computed for each foot and an average stepping frequency was obtained for each participant. The stepping frequency variance of each participant was defined as the variability of the stepping frequency over the trial. This was calculated by splitting the condition into 10 s segments and calculating a stepping frequency for each segment. The standard deviation of these stepping frequencies over all segments for each patient and each condition was then used to quantify variability. For analyses focusing on the stepping frequency variability during the first and last parts of the condition, the stepping duration of each step was recorded, and the standard deviation was calculated separately for the first 20 s and the last 20 s.

#### LFP and EMG analysis

CWT was used for time-frequency decomposition of the chosen bipolar LFPs in the STN and the EMG activities from the recorded muscles. The data was pre-processed with a 100 Hz low pass filter, a 1 Hz high pass filter (both second-order, two-pass Butterworth filters) and a 50 Hz notch filter to eliminate line noise. CWT was used for time-frequency decomposition with a Morlet wavelet of 10 cycles and a standard deviation of 3. The amplitude of each frequency band at different time points was calculated by taking the absolute value of the complex output. The average amplitude for different frequency bands and task conditions were then calculated. The amplitude of each individual frequency (per 1 Hz) was *Z*-scored over each condition (ON and OFF) for each participant and then mean averaged over the period and frequency band in question. In this study, beta band activity was defined within the frequency range of 12–35 Hz,^40,41^ with low-beta activity defined as 12–20 Hz and high-beta activity defined as 21–35 Hz. This broad range is selected as stepping and lower limb movements seem to be associated with modulation of activities over a broader frequency band extending beyond 30 Hz.^12,27,42^

#### STN beta burst analysis

Beta bursts were defined as time periods where the average beta amplitude exceeded its 75th percentile for a minimum of 200 ms.^43,44^ This 75th percentile was calculated for each condition separately, meaning there were differing raw thresholds for bursts.

#### Intermuscular coherence and STN-muscle coherence

The phase locking value (PLV)^45,46^ was used to calculate STN-muscle coherence and intermuscular coherence (IMC). This was to compute the phase consistency between the STN and the lower extremity muscles, as well as the IMC between these muscles. The PLV provides estimates of synchrony independent of the amplitude of oscillations. This is in contrast to measures of coherence where phase and amplitude are intertwined.^47^ To calculate PLVs, the signals of interest were first band-pass filtered using a digital IIR filter, prior to Hilbert transformation. The instantaneous phase of each signal at each time point was extracted, and the phase differences between the signals were calculated. The vector strength of the phase difference was computed using a sliding window technique with a fixed window length of 250 ms period, with 125 ms before the sample and 125 ms after. The value at each time point is the vector strength of the phase difference over this 250 ms window. This was computed over the entire duration of the condition under analysis. This procedure was repeated for each frequency band to generate a time-frequency coherence plot. The mean was found for each patient, before finally computing the mean across all patients.

For STN-muscle coherence, the STN signal was kept the same and the EMG data was randomly shuffled to generate a comparison which was subtracted from the original to give the difference between the observed data and the shuffled data. This eliminates the influence of one signal and focuses on the coherence between the two of them.

#### Stepping phase-related modulations

To analyse the stepping phase-related changes in the STN LFPs, EMG activities, as well as the STN-muscle connectivity and IMC, the time series of the recorded stepping force of each foot was first Hilbert transformed to find the phase of the step, from −π/2 to π/2 radians. Each step cycle was then divided into 181 different bins according to the calculated phase. The average amplitude of STN LFP activities, muscle EMG activities, STN-muscle connectivity and IMC in different frequency bands were found for each phase bin. These were *Z*-scored for each participant, by condition (ON and OFF), as described in the ‘LFP and EMG analysis’ section. This result was then averaged over all patients, allowing for analysis of electrophysiological modulation according to stepping phase.

#### Trial rejection

Prior to processing in MATLAB, the raw recorded data was loaded into Spike2 for visualization. Obvious artefacts were identified and only data considered as clean was selected for inclusion in the analysis. Artefacts include ocular, jaw clenching or mechanically induced (from the cable movement) disturbances on the salient channels for the analysis, particularly the STN LFPs and EMG data. In addition, clearly identifiable stepping-induced force readings from the plates was also required to define data from that step as clean. This procedure resulted in an average of 14.22% *±* 21.98% of data excluded from ON medication, and 15.66% *±* 23.31% excluded from OFF medication, with the value for each patient given in Table 1.

#### Statistics

Due to the small sample sizes available, non-parametric tests were used to increase the robustness of the results. When analysing the effects of two or more experimental conditions (for example medication, frequency band and movement status) a non-parametric approach was used by pre-processing the data with the Aligned Rank Transform before applying a repeated measures ANOVA. The Wilcoxon Signed Rank test was used for *post hoc* testing, and when there was only one experimental condition with two groups.

Furthermore, permutation-based cluster analysis was utilized to test whether the EMG amplitude, STN-muscle coherence and IMC in different frequency bands during stepping were significantly different between ON and OFF levodopa medication, or between beta bursts and no bursts. This was implemented by generating a paired *t*-test plot between the two conditions; *t*-statistics for the clusters were then determined, as well as the sizes of the clusters in pixels, with the largest values selected as the largest cluster. Then, the null distribution was generated by randomly swapping the pairings in the paired samples *t*-test, where the largest *t*-statistic and cluster size were recorded for each permutation, which created a set of possible cluster sizes under the null hypothesis. Finally, two *P*-values were generated, one for the cluster size and one for the accumulated *t*-statistic, computed by comparison with the null distribution. Only the overall *P*-value from the accumulated *t*-statistic method is reported here, because there were no results that changed between the two methods.

## Results

### Levodopa decreases variance of patient stepping frequency

**Figure 1:**
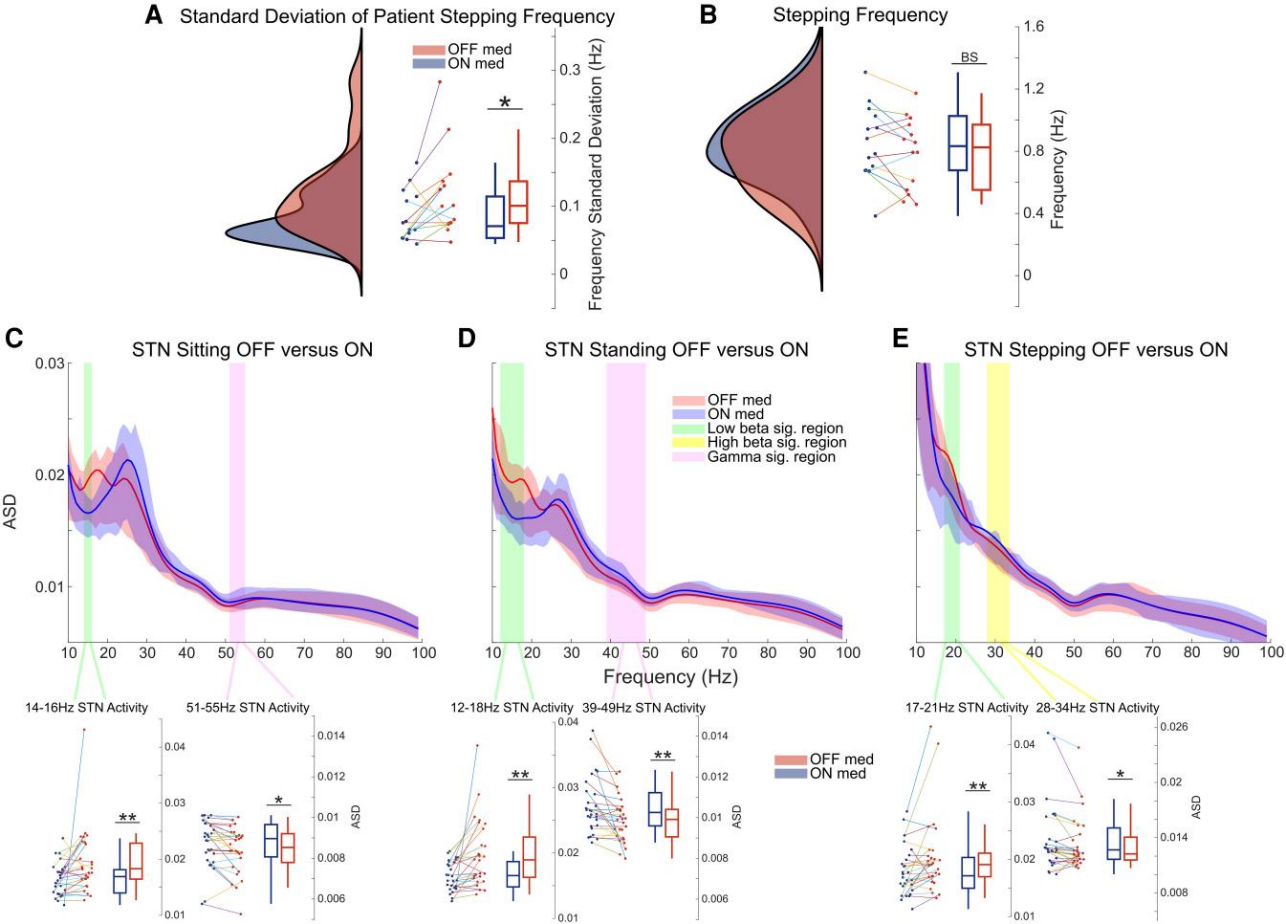
**Behavioural results and medication-related changes in STN LFPs in different movement states**. A box plot of the standard deviation of the patients’ stepping frequency is presented in (**A**) while the patients’ stepping frequency is depicted in (**B**) (where BS is borderline significant). The amplitude spectral density (ASD) of activity in the STN over the sitting, standing and stepping trials is presented in **C**, **D** and **E**, respectively, where the shaded area is the 25th to 75th percentile. The box plots below **C**, **D** and **E** demonstrate the group median, 5th, 25th, 75th and 95th percentile, as well as the data for each participant (for each STN), for the significant cluster. LFP = local field potential; STN = subthalamic nucleus.

To evaluate behavioural changes induced by medication, analyses were conducted on patient stepping patterns (i.e. stepping frequency and its variability). Results indicated that there was a borderline significant effect of medication on the stepping frequency, with a marginal increase in the frequency when ON medication (*Z* = −1.73*, P =* 0.084; Fig. 1B). Furthermore, an analysis of the variability (standard deviation) of stepping frequency revealed a statistically significant difference between conditions (*Z =* 2.29*, P =* 0.022; Fig. 1A), with reduced variability in stepping frequency when ON medication.

A two-way ANOVA was conducted to evaluate the effects of medication (ON versus OFF) and time-related changes on stepping frequency and its variability, focusing on the initial versus final 20 s intervals of stepping. This tested whether the overall increase in step frequency variability when OFF medication was due to changes over time. This analysis showed that medication had a significant effect on stepping frequency [*F*(1,39) *=* 4.18, *P =* 0.048], increasing it on average. It also showed a statistically significant effect of the time interval [*F*(1,39) *=* 7.70, *P =* 0.008], with stepping frequency increasing in the final 20 s. For variability, there was an effect of medication on the standard deviation of stepping frequency in the initial and final 20 s of stepping [*F*(1,39) *=* 5.55, *P =* 0.024]. However, the time interval did not affect the stepping frequency standard deviation [*F*(1,39) = 0.63, *P =* 0.43] and no interaction effect was observed with medication [*F*(1,39) = 0.18, *P =* 0.67]. This implied that the variability in stepping frequency was not due to time-related changes (such as slowing down or speeding up).

### Different effects of levodopa and locomotor status on low- versus high-beta band activities in STN LFPs

Initial analysis examined the effects of medication on the amplitude spectral density (ASD) of STN LFPs recorded during sitting, standing and stepping, using a permutation cluster analysis, with results presented in Fig. 1C–E. The ASD was computed for frequencies ranging from 10 to 100 Hz and normalized by dividing each value by the area under the curve. The cluster-based permutation test showed that medication reduced activities in the low-beta frequency band across all different movement states, even though the exact frequency band is slightly different (Fig. 1C–E). During resting, this analysis revealed significant reduction in the activities between 14–16 Hz with medication. During stepping, this analysis revealed a significant reduction in the 17–21 Hz band (*t =* 2.66, *P =* 0.011), as well as an increase in 28–34 Hz activity (*t = −*2.30, *P =* 0.017) when ON medication compared with OFF medication. During standing, levodopa medication also decreased the activity in the 12–18 Hz band (*t =* 3.92, *P <* 0.001) while increasing activity in 39–49 Hz (*t* = −3.46, *P <* 0.001).

Subsequently, a three-way ANOVA was conducted on the beta amplitude with main factors of medication (ON versus OFF), movement status (sitting versus stepping versus standing) and sub-beta frequency band [high (21–35 Hz) versus low (12–20 Hz)] with the frequency ranges frequently used in previous publications. This analysis revealed that there were significant main effects of medication [*F*(1,297) = 4.39, *P =* 0.037], movement status [*F*(2,297) = 4.85, *P =* 0.008] and beta frequency band [*F*(1,297) = 111.14, *P <* 0.0001], on the different sub-beta amplitudes. There was a significant interaction between medication and beta frequency band [*F*(1,297) = 13.14, *P =* 0.0003], and a significant interaction between movement status and beta frequency band [*F*(2,297) = 26.79, *P* < 0.0001]. However, there was no interaction between medication and movement status [*F*(2,297) = 0.14, *P =* 0.87], nor three-way interaction between medication, movement status and beta frequency band [*F*(2,297) = 0.11, *P* = 0.90].

**Figure 2:**
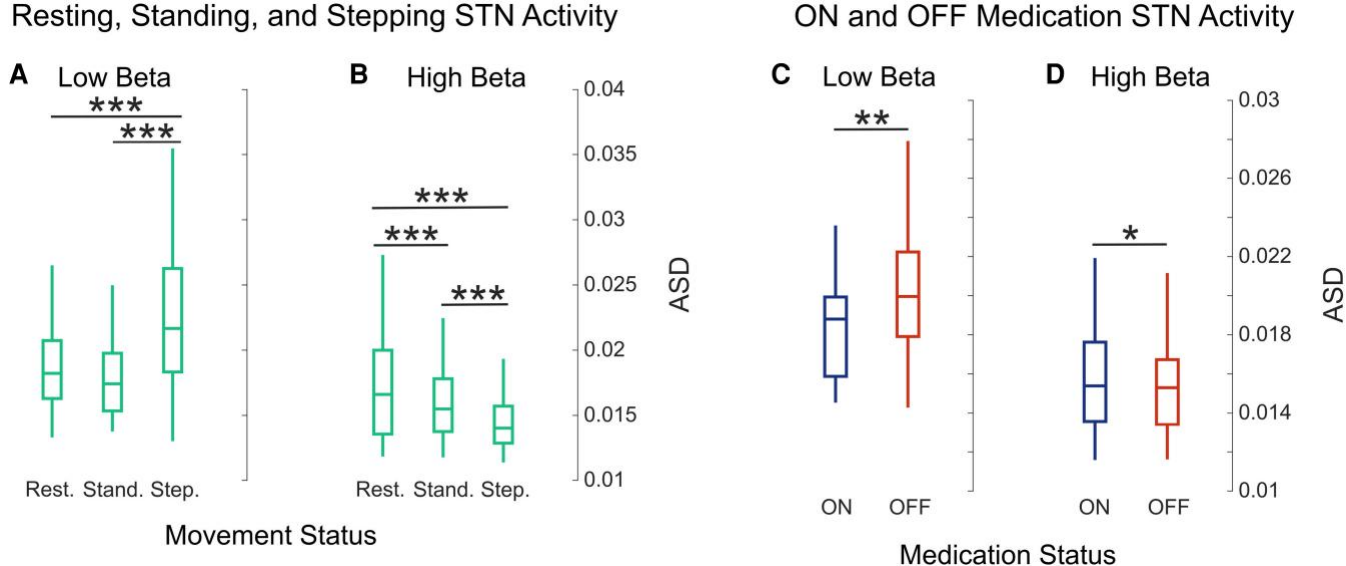
**STN activity by movement and medication status.** A box plot displaying amplitude spectral density (ASD) for resting, standing and stepping, averaged over medication status is displayed in (**A**) for low-beta and (**B)** for high-beta. The medication status (ON versus OFF) averaged over the movement status (resting, standing, stepping) is presented in (**C**) for low-beta and (**D**) for high-beta. STN = subthalamic nucleus.

Further non-parametric ranked tests applied to data collapsed across medication status were used to explore the two-way interaction between the locomotion status and the sub-beta frequency bands (Fig. 2A and B). For high-beta, this showed that there was a significant difference between sitting and standing (*Z =* 3.80, *P =* 0.0001), sitting and stepping (*Z =* 4.12, *P <* 0.0001), and between standing and stepping (*Z =* 4.01, *P <* 0.0001). Additionally, for low-beta, there was no significant difference between sitting and standing (*Z =* 0.75, *P =* 0.45), but there was between sitting and stepping (*Z = −*3.39, *P =* 0.0006), and standing and stepping (*Z = −*4.01, *P <* 0.0001). Overall, stepping was associated with the highest low-beta band activities and the lowest high-beta activities compared with standing and sitting. The results were corrected for multiple comparisons utilizing the Bonferroni correction with a significance threshold of 0.05 divided by 6 (0.008).

Non-parametric ranked test applied to data collapsed across locomotion status was used to further explore the significant two-way interaction between medication and sub-beta frequency bands (Fig. 2C and D). This revealed significant differences for low-beta (*Z* = −2.98, *P* = 0.003) and for high-beta (*Z* = 2.28, *P* = 0.023), where medication reduced the activities in the low-beta frequency bands and increased the activities in the high-beta frequency band, when considering all data averaged across different movement states. The *post hoc* tests utilize the Bonferroni correction for multiple comparisons with a significance threshold of 0.05 divided by 2 (0.025).

### Reduced beta-band activity around the contralateral late stance and lift-off phase when ON levodopa

**Figure 3:**
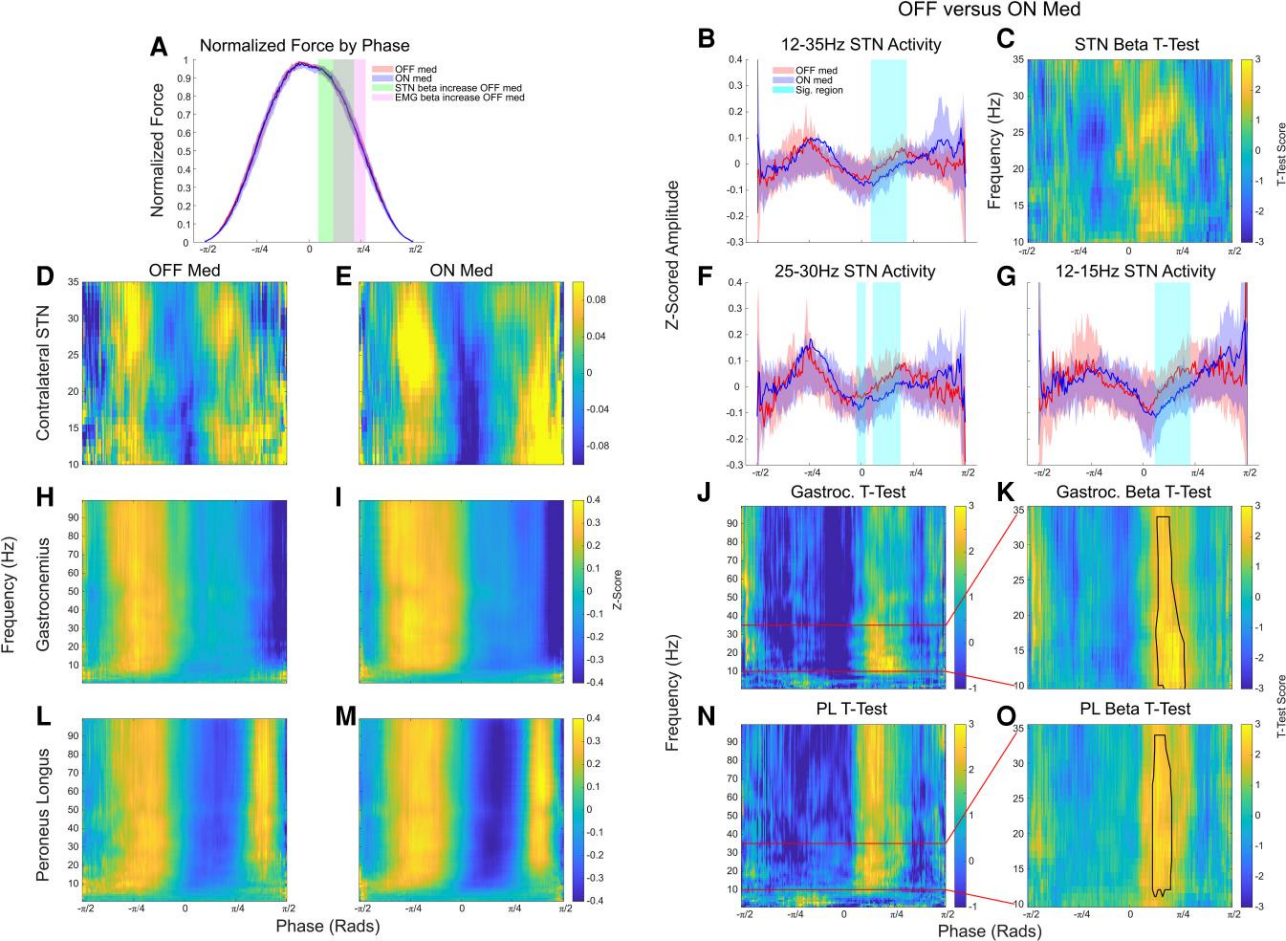
**Analysis of electrophysiology by stepping phase**. The normalized force of the plates by the stepping phase, computed using the Hilbert transform, is depicted in **A**. The time frequency decompositions of the STN, gastrocnemius and peroneus longus OFF medication are displayed in **D**, **H** and **L**, while ON medication items are displayed in **E**, **I** and **M**, respectively. Data are normalized within condition, i.e. separate normalization for each condition. **B**, **F** and **G** depict the beta activity averaged over 12–35 Hz (**B**), 25–30 Hz (**F**) and 12–15 Hz (**G**), for OFF and ON medication, while the *t*-test scores between OFF and ON in the time-frequency domain in the STN, gastrocnemius and peroneus longus are shown in **C**, **K** and **O**, respectively. The *t*-test scores between OFF and ON from 1 to 100 Hz are presented in **J** for the gastrocnemius, and **N** for the peroneus longus. Statistically significant clusters are outlined in **K** and **O** for gastrocnemius (*P* = 0.030) and peroneus longus (*P* = 0.043), respectively. STN = subthalamic nucleus.

To explore the effect of stepping phase on STN and EMG activity, and whether medication induced phase-specific changes, force plate readings were used to extract the step phase, as detailed in the ‘Stepping analysis’ section (Fig. 3A). Consistent with previous findings,^12^ step-phase-related modulation of beta band activities in the STN LFPs were observed. These modulations mirrored contralateral muscle activity both ON and OFF medication (Fig. 3D, E, H, I, L and M), with beta band activities in the STN LFPs increasing and decreasing in sync with muscle activities in the contralateral leg. One-dimensional permutation cluster analysis demonstrated a significant difference in STN beta activity (12–35 Hz) from 0 to π/4 (the late stance phase after peak stepping force) between OFF and ON medication states (*t =* 2.299, *P* = 0.018; Fig. 3B). This phase corresponds to weight-shifting and movement initiation (lift-off) stages of the gait cycle, where beta activity is generally reduced. This beta reduction was more pronounced in the ON medication state, indicating a stronger suppression of beta activity during the contralateral late stance and lift-off phase compared with the OFF medication state. The same analysis was then conducted for the EMG activity, which revealed a clear increase in muscle activity during the same 0 to π/4 phase in both muscles (gastrocnemius: *P =* 0.030, peroneus longus: *P* = 0.043) when OFF medication compared with ON medication (Fig. 3J, K, N and O). While this increase of activity was observed across beta and into the gamma range, the exaggerated beta activity was consistent across muscles and particularly prominent in the gastrocnemius.

### Increased beta-band STN-muscle and intermuscular coherence during late stance phase

**Figure 4:**
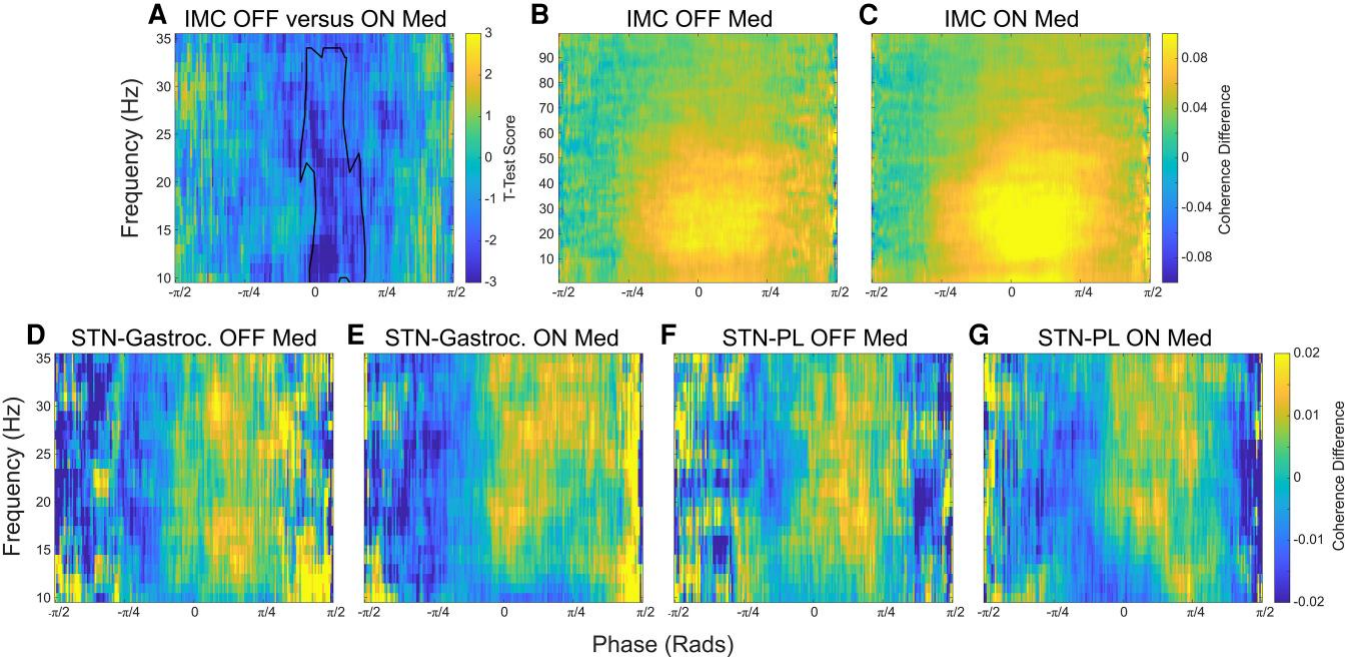
**Intermuscular and STN-EMG coherence by stepping phase.** (**A**–C) Intermuscular coherence (IMC) by stepping phase between the gastrocnemius and peroneus longus muscles of the same leg. (**D** and **E**) STN-gastrocnemius coherence by stepping phase, and (**F** and **G**) STN-peroneus longus coherence. (**B**, **D** and **F**) Time-frequency maps for OFF medication and (**C**, **E** and **G**) ON medication. The difference between OFF and ON medication for IMC (based on the *t*-test score) is presented in **A**, and statistically significant clusters are outlined in black (*P* = 0.020). For **B–G**, the coherence difference is computed by first calculating the phase locked coherence and then subtracting a permuted version where the phase locked coherence is recalculated but with permuted EMG signal (for IMC only one of the signals is permuted). STN = subthalamic nucleus.

Further analysis examined STN-muscle coherence and IMC (between the gastrocnemius-peroneus longus coherence of the same leg) in relation to the stepping phase. The step-phase-related modulation pattern of the IMC (Fig. 4B and C) showed an opposite pattern compared with the muscle amplitude modulation pattern (Fig. 3H, I, L and M). The STN-muscle coherence and IMC in the alpha/beta bands increased from phase 0 to π/4 (i.e. the late stance and lift-off phase), while amplitudes of STN and muscle activities decreased relative to phases in the step cycle. Permutation cluster analysis revealed a significant increase in IMC in the beta band (*P =* 0.020) between 0 and π/4 when ON medication compared with OFF (Fig. 4A). This indicates that when OFF levodopa, there was reduced phase synchrony between the gastrocnemius and peroneus longus muscles in the same leg during late stance and lift-off phase compared with ON medication, despite the overall increase in muscle activity in the same time window.

### STN beta bursts are linked to increased beta band activities in the EMG

**Figure 5:**
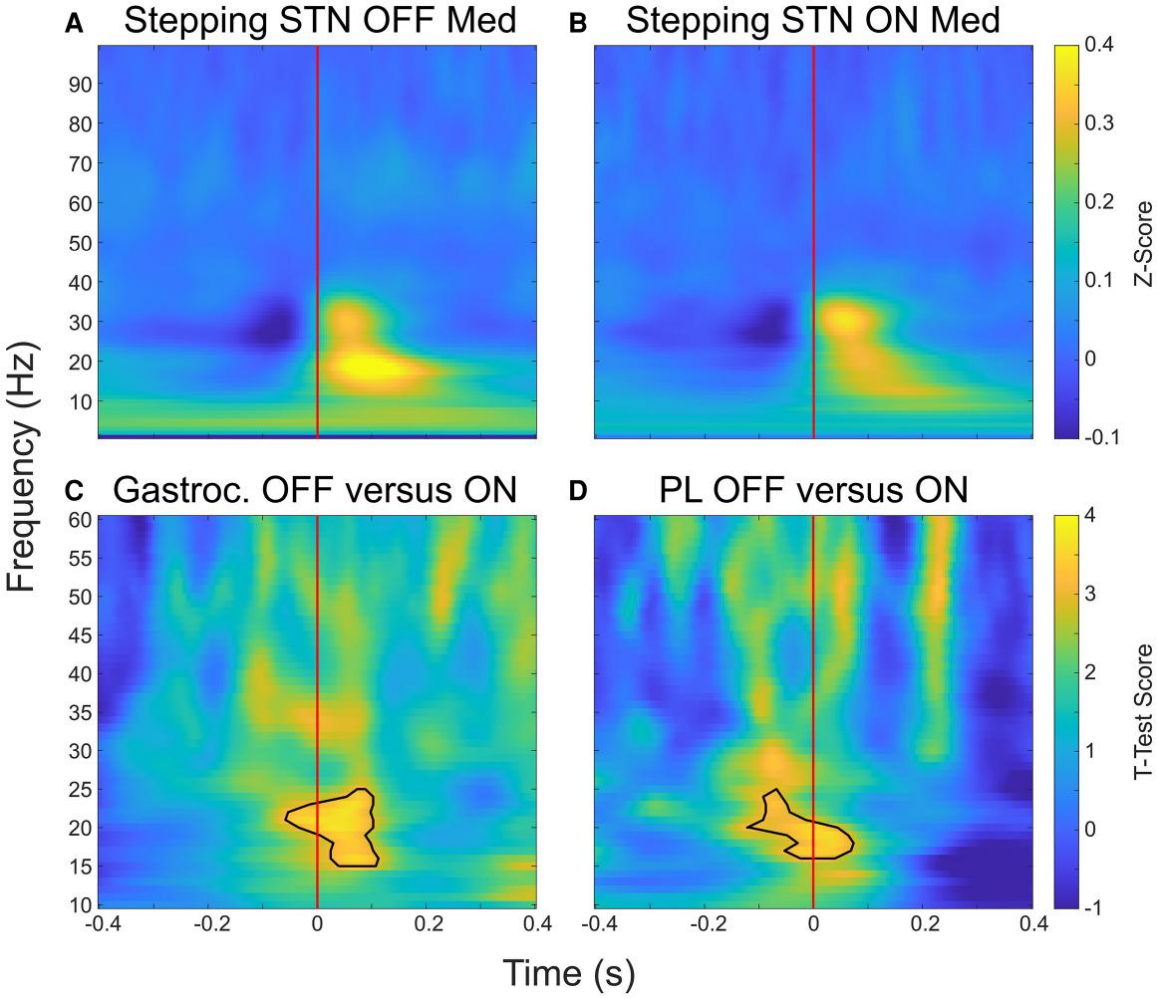
**Effect of STN beta bursts from phases 0 to π/4.** Beta bursting activity is extracted where the amplitude exceeds the 75th percentile (indicated by the vertical red line in the figures), shown for OFF medication (**A**) and ON medication (**B**). The concomitant activity is selected for the gastrocnemius and peroneus longus, with the *t*-test score between OFF medication and ON medication computed and presented in **C** and **D**, respectively. Statistically significant clusters are outlined in black for gastrocnemius (*P* = 0.020) and peroneus longus (*P* = 0.040). STN = subthalamic nucleus.

Beta bursting activity was extracted according to the procedure outlined in the ‘STN beta burst analysis’ section. In addition, the amplitude by frequency of the EMG was computed over the entire recording. Then, EMG activity corresponding to STN beta burst onset was extracted. Due to the previous finding showing phase-locked increases in EMG activity during a specific phase of the stepping cycle, only STN bursts occurring between 0 and π/4 radians were considered, presented in Fig. 5A and B. A permutation cluster analysis revealed a significant increase in EMG beta band amplitudes around STN beta burst onset between OFF and ON medication for both the gastrocnemius (*P =* 0.020; Fig. 5C and the peroneus longus (*P =* 0.040; Fig. 5D).

## Discussion

This study provides key insights into the role of beta oscillations during stepping in PD and the modulatory effects of levodopa. Firstly, levodopa medication and movement status were found to have opposing effects on low- versus high-beta activity, indicating different roles of these beta sub-bands in motor control in PD. Secondly, a step-phase-specific effect of levodopa on beta oscillations during stepping was observed, with significantly decreased beta activity in the STN, the gastrocnemius, and the peroneus longus muscles during the late stance and lift-off phase when ON levodopa medication. Lastly, STN beta bursts during this stepping phase were associated with increased beta activity in the EMGs in the gastrocnemius and peroneus longus muscles. Increased beta activity in the STN and lower extremity muscles may be associated with reduced movement efficiency, increased exertion and impaired motor coordination. Together, these findings emphasize the importance of phase-specific beta modulation and its relationship with muscle activation, providing a potential rationale for targeted phase-based stimulation strategies to address gait impairments in PD.

### Different effects of movement status on average amplitude of low- versus high-beta frequency bands

The results demonstrate opposing effects of movement status (stepping versus standing and stepping versus resting) on the average amplitude of low- versus high-beta frequency bands in the STN. Specifically, stepping was associated with increased low-beta and reduced high-beta activity compared with standing and resting, independent of medication status. Suppression of high-beta might be necessary during stepping to facilitate active alternating movements, such as lifting, swinging and lift-off. This is consistent with a previous study showing that lower limb movements were associated with greater desynchronization in the high-beta frequency bands (24–31 Hz) compared with low-beta band or upper limb movements.^27^ Meanwhile, an earlier study has shown that PD patients with FOG exhibited elevated high-beta power compared with those without FOG, with significant reductions in high-beta power following levodopa administration along with suppression of FOG.^24^

The present findings also reveal different effects of levodopa on low- versus high-beta frequency bands in the STN. When averaged across all movement states (resting, standing and stepping), levodopa reduced the amplitude of low-beta frequency band oscillations while increasing high-beta oscillations. The reduction in low-beta activity is consistent with previous findings,^8,48,49^ highlighting its pathological nature. On the other hand, the increase in high-beta activity suggests a potential physiological function of this frequency band during resting, standing and stepping. Recent research supports the notion that supplementary motor area activity selectively drives high-beta STN activity via the hyperdirect pathway, suggesting a functional role for high-beta frequencies in cortico-subcortical communication.^26^ Furthermore, low- versus high-beta band cortico-subcortical coherence have been implicated to play different roles in movement inhibition and expectation.^50^

It should be recognized that most patients in this study were tremor dominant or exhibited main symptoms of bradykinesia and rigidity, hence, comparisons between patients with and without FOG were not feasible. Future studies focusing on patients with FOG may further elucidate the pathophysiological role of low- versus high-beta in gait impairment in PD.

### Step-phase-specific effect of levodopa on STN LFPs, EMGs and IMC during stepping

Consistent with previous findings, beta band activity in the STN exhibited step-phase-related modulation, increasing during the early stance phase and decreasing during the late stance phase and lift-off of the contralateral leg. This reduction in beta activity during the late stance and lift-off phase may be linked to movement initiation.^12,51,52^ Yet, the role of muscle activity during stepping is less understood. In the present study, medication increased the IMC during the late stance and lift-off phase, despite a reduction in total amplitude of muscle activity, which may indicate more effective coordination between the recorded muscles during movement initiation. This is consistent with a previous study showing that patients with PD exhibited reduced intermuscular zero-lag coherence in the beta/gamma frequency band during the end-of-stance phase.^53^

In addition, a step-phase-specific effect of levodopa on STN LFPs, EMGs and IMC during stepping was observed, which was constrained to the late stance and lift-off phase. When OFF medication, there was an increase of STN beta activity and EMG activities, as well as reduced IMC in the affected phase. Increased STN beta activity during this phase window may be associated with delayed movement initiation and reduced movement speed.^54-56^ On the other hand, increased EMG activities in both the gastrocnemius and peroneus longus were observed, but with a reduction of intermuscular coherence (phase synchrony) in the beta frequency band. The reduction in STN beta activity appears to precede changes in EMG activity (Fig. 3A), suggesting a causal relationship where STN activity influences muscle activation. This is further supported by the observed effect of beta bursts on EMG activity, which revealed an increase in muscle activation following heightened beta activity in the STN, similar to previous observations of cortical beta bursts.^32,33^ Conversely, reduction of STN beta activity could enable the muscle to release from a tonic state and transition more smoothly into the step phase.

Although the increase in muscle activity resulting from STN beta bursts may initially seem minor, it may have significant consequences for gait. One possible outcome is a reduction of movement efficiency, requiring greater effort to complete the same movement.^57^ This could result in faster depletion of energy levels,^58^ further slowing movement and impairing motor coordination. Increased muscle activity may also inhibit natural stepping motion, which may result in compensatory mechanisms and strategies that are suboptimal,^59^ such as recruitment of other muscle groups which could also reduce energy levels.^60^ Furthermore, this increase of muscle activity with reduced intermuscular coherence indicates altered muscle activation patterns, which are both linked to altered timing of the gait cycle.^61^ In turn, this alteration of timing has been proposed as a possible contributor to FOG.^30^ Similarly, the inability of patients to effectively produce muscle coordination at movement initiation may also contribute to stepping disturbance. This could result in a number of consequences, including an increase in effort to execute the movement, prompting of compensatory mechanisms and higher levels of fatigue.

### Implications for future stimulation strategies

There are several implications for future stimulation strategies that emanate from this work. The results suggest that during parkinsonian stepping the motor outputs (both beta band activities in the STN LFPs and EMG activities in the lower extremity) were significantly affected from phases 0 to π/4 in each step cycle, which correspond to the late stance and lift-off phase. This supports the view that stimulating between 0 and π/4, utilizing a stepping-based phase-triggered stimulation strategy, could further probe the causal relationship between the observed increase in STN beta band activity and gait impairment in PD. It may also offer beneficial effects for patients during stepping and potentially free walking. Additionally, it could reduce variations of timing in the gait cycle, alleviating one of the contributing factors to FOG, thereby potentially reducing these episodes. Another possible ramification is a reduction in reliance on compensatory mechanisms.

### Limitations and future work

A primary limitation of this work, due to the challenge of recruiting patients with externalized leads, is the limited sample size. Another limitation is that stepping-in-place was recorded for this study in order to minimize movement-related artefacts in the LFP and EMG recordings. Stepping-in-place is different from free walking, for instance, there is no forward momentum during stepping-in-place (which is crucial for walking). Moreover, the stepping-in-place task involves vertical up and down leg movements while maintaining balance rather than propelling the body forward as during walking. Some participants reported that stepping-in-place is less fluid or automatic than walking. Therefore, attributing the present effects directly to free walking should be approached cautiously, although a previous study showed similar pattern of STN beta-band modulation aligned to the stepping phase within each step cycle during free walking in a few patients.^12^ Moreover, analyses in this study focused on steps that could be reliably detected using force plate measurements and did not include episodes of hastening or true freezing. Whether the observed increases in STN beta activity and intermuscular coherence during the late stance phase when OFF levodopa are associated with poor motor performance during free walking remains to be tested in future studies. Additionally, while the gastrocnemius and peroneus longus were selected in this study for their known roles in stance stability and foot control, simultaneous monitoring of multiple muscles in future studies, especially multiple antagonistic muscle pairs, such as the gastrocnemius and tibialis anterior may offer more insight on the pathophysiology of free walking. Finally, the timing of experimental sessions may represent a confound, as the OFF medication sessions are recorded in the morning and the ON medication sessions in the afternoon, and stepping was recorded after standing in both medication conditions. Patients may have experienced reduced energy and motivation later in the day, potentially affecting performance.

Future work could focus on STN activity around periods that can instigate freezing episodes for example, stopping, turning or moving through a doorway. Furthermore, studies testing different stimulation strategies at different phases of stepping are critical to evaluate the efficacy of the proposed approach, with testing in real-world gait scenarios a prerequisite for clinical translation.

## Supplementary Material

awaf464_Supplementary_Data

## Data Availability

All data will be shared on the MRC BNDU Data Sharing Platform (https://data.mrc.ox.ac.uk/) upon publication.
