# Supplementary material for: Dopamine and the dynamics of subthalamic and leg muscle activities in parkinsonian stepping
Source: Brain. 2025 Dec 13;149(7):2409–21. doi: 10.1093/brain/awaf464 (PMC13337239; doi:10.1093/brain/awaf464)
Supplement: awaf464_Supplementary_Data [file awaf464_supplementary_data.pdf]

Supplementary Figures

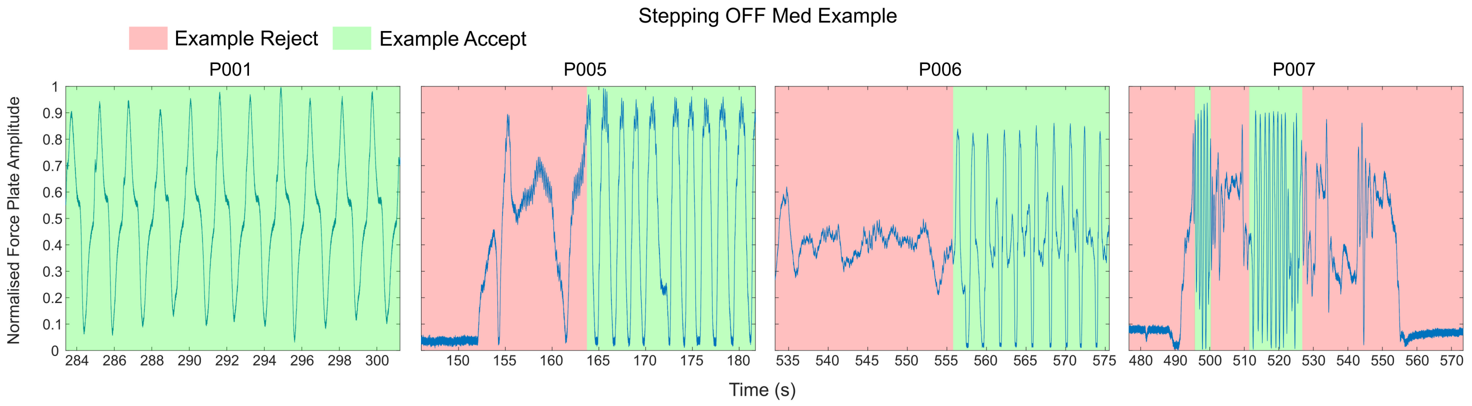

Supplementary Figure 1: Examples of normal stepping data (highlighted in green) and rejected data (red) based on force plate measurements.

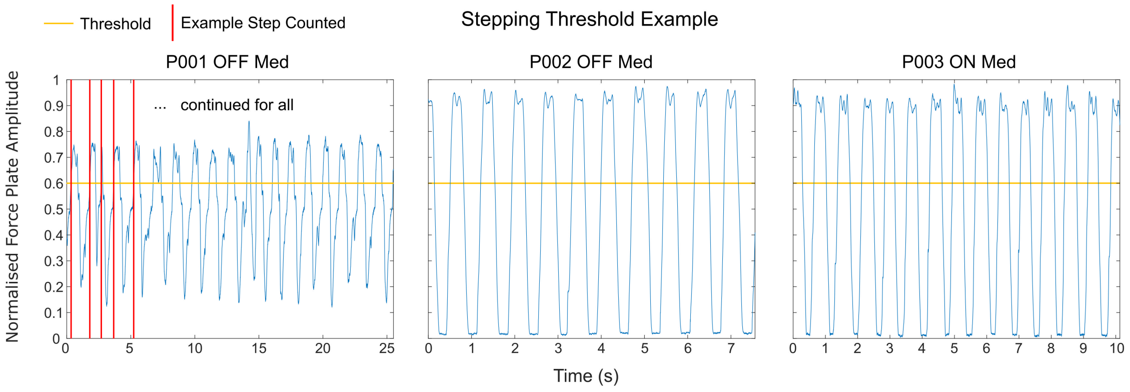

Supplementary Figure 2: Stepping threshold examples. Examples of how steps were counted. The threshold was selected visually based on the data. The horizontal yellow line denotes the threshold used, while the vertical red line indicates a step counted when the amplitude exceeds the chosen threshold.

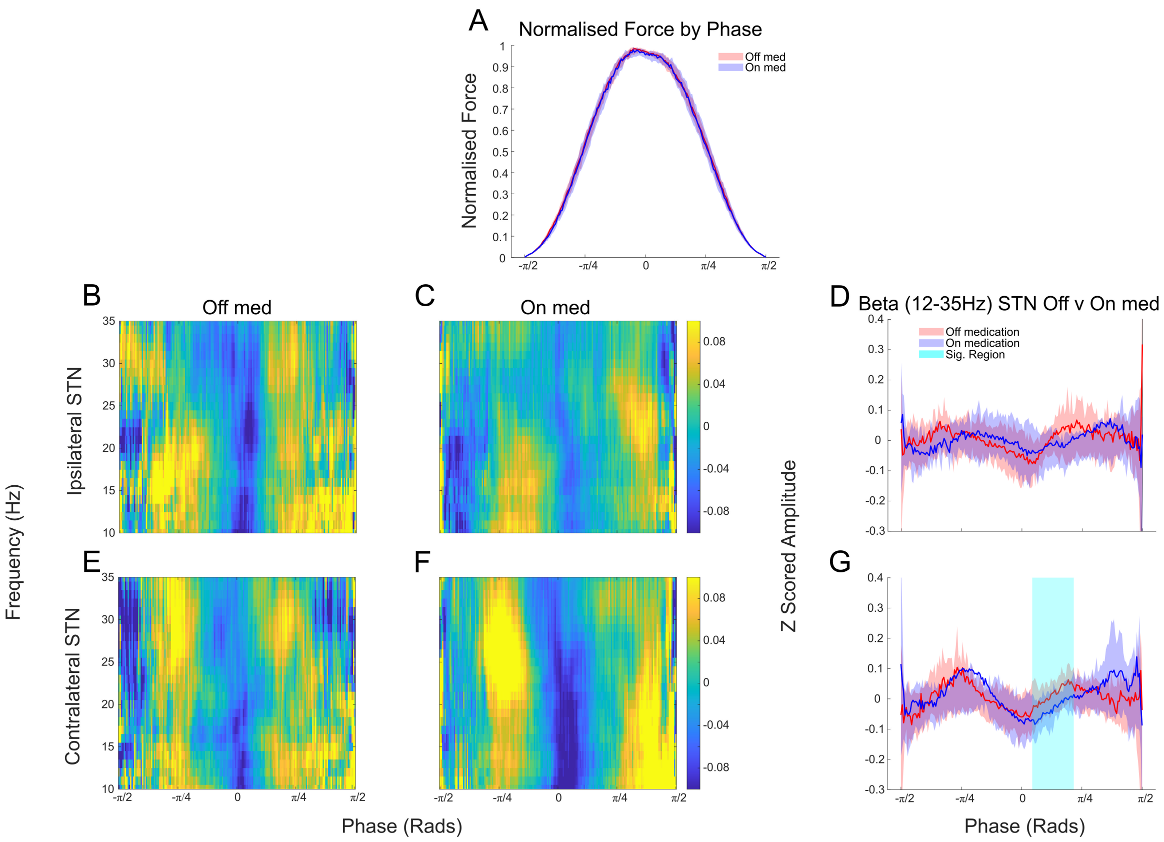

Supplementary Figure 3: Ipsilateral STN and contralateral STN power spectra changes aligned to step phase. A demonstrates the normalised force by phase, while **B** shows the ipsilateral STN activity while OFF medication, **C** shows it ON medication, and **D** shows the z-scored amplitude of the ipsilateral STN in both OFF and ON conditions. **E** depicts the contralateral STN activity while OFF medication, **F** shows it while ON medication, and **G** depicts the z-scored amplitude of the contralateral STN in both OFF and ON conditions.
